# Mass Spectrometry in Cerebrospinal Fluid Uncovers Association of Glycolysis Biomarkers with Alzheimer’s Disease in a Large Clinical Sample

**DOI:** 10.21203/rs.3.rs-3073597/v1

**Published:** 2023-06-27

**Authors:** Matthijs B. de Geus, Shannon N. Leslie, TuKiet Lam, Weiwei Wang, Pia Kivisakk, Angus C. Nairn, Steven E. Arnold, Becky C. Carlyle

**Affiliations:** Massachusetts General Hospital; Yale School of Medicine; Yale School of Medicine; Yale School of Medicine; Massachusetts General Hospital; Yale School of Medicine; Massachusetts General Hospital; Kavli Institute for Nanoscience Discovery

**Keywords:** Alzheimer’s disease, Cerebrospinal fluid, Glucose metabolism, Biomarkers, Mass spectrometry

## Abstract

**Background:**

Alzheimer’s disease (AD) is a complex heterogenous neurodegenerative disorder, characterized by multiple pathophysiologies, including disruptions in brain metabolism. Defining markers for patient stratification across these pathophysiologies is an important step towards personalized treatment of AD. Efficient brain glucose metabolism is essential to sustain neuronal activity, but hypometabolism is consistently observed in AD. The molecular changes underlying these observations remain unclear. Recent studies have indicated dysregulation of several glycolysis markers in AD cerebrospinal fluid and tissue.

**Methods:**

In this study, unbiased mass spectrometry was used to perform a deep proteomic survey of cerebrospinal fluid (CSF) from a large-scale clinically complex cohort to uncover changes related to impaired glucose metabolism.

**Results:**

Two glycolytic enzymes, Pyruvate kinase (PKM) and Aldolase A (ALDOA) were found to be specifically upregulated in AD CSF compared to other non-AD groups. Presence of full-length protein of these enzymes in CSF was confirmed through immunoblotting. Levels of tryptic peptides of these enzymes correlated significantly with CSF glucose and CSF lactate in matching CSF samples.

**Conclusions:**

The results presented here indicate a general dysregulation of glucose metabolism in the brain in AD. We highlight two markers ALDOA and PKM that may act as potential functionally-relevant biomarkers of glucose metabolism dysregulation in AD.

## Introduction

Alzheimer’s disease (AD) is the most prevalent form of dementia affecting over 6 million people in the United States in 2022 ^1^. The essential pathological findings of AD in the brain are amyloid-beta (Ab) plaques and paired helical filaments of hyperphosphorylated tau (PHFtau) which reflect a disorder of proteostasis encompassing protein misfolding, aggregation and/or impaired clearance ^2^. However, the causes and consequences of AD pathology are complex with varying degrees of inflammation ^3^, neurovascular function ^4^, and brain metabolism ^5^ driving the dysproteostasis that contributes to neurodegeneration and resultant dementia. The development of biomarkers of AD has become essential not only for accurate diagnosis during life, but also for better understanding the various pathophysiological factors that contribute to disease emergence and progression. As the defining pathological features of AD, measurements of Ab and tau proteins have now been established as core biomarkers for AD diagnosis via PET imaging or biochemical assays in cerebrospinal fluid (CSF) and blood ^6,7^. However, given the multi-pathophysiological basis of pathology, neurodegeneration and clinical dementia of AD and commonly comorbid pathologies such as cerebrovascular disease and diabetes, it is important to develop a wider set of biomarkers, beyond Ab and tau ^8^.

Current clinical biomarkers are measured in CSF or through functional and structural neuroimaging such as MRI and PET. CSF is in continuous exchange with the interstitial fluid of the brain and is enriched with a host of proteins secreted, excreted or otherwise released from neurons and glia. Thus, CSF remains the most accessible and informative matrix to measure biochemical changes occurring in the brain, whereas fluids like blood plasma struggle with low analyte concentration of neuronal and glial proteins diluted in a vast mix of proteins from organs throughout the periphery ^9^.

Glucose is the primary fuel that powers the vast synaptic activity in the human brain and abnormalities in glucose metabolism measured by fluorodeoxyglucose positron emission tomography (FDG-PET) correlate with distortions of synaptic activity in AD ^10–12^. Neurons require efficient metabolism of glucose through glycolysis and oxidative phosphorylation ^12,13^. Alternatively, in events of brain injury, lactate can be used as an alternative source of energy ^12,14^. Abnormalities in regional brain glucose utilization in AD, as measured by FDG-PET, have been recognized for decades and evidence of neuronal insulin resistance, as measured in postmortem tissue research, is also well described ^15–17^. Additionally, one of the major risk factors for AD is type 2 diabetes mellitus, a disease characterized by insulin resistance, but the biological nature of this association is still unclear. Multi-omic studies have shown changes in several markers of glucose metabolism in both CSF and tissue from AD patients (Higginbotham et al., 2020; Johnson et al., 2020). Recently, Traxler *et al.,* (2022), reported a shift from aerobic to anaerobic glucose metabolism in an induced neuronal model of AD, indicating functional metabolic changes occurring within neurons in AD.

Despite an identified association between altered brain glucose metabolism and AD, specific mechanistic biomarkers of altered glucose metabolism in AD have not been identified. Unbiased screening of protein and/or peptide levels in CSF, in relation to changes of the traditional Ab and tau biomarkers in a clinically diverse cohort, could help identify distinct markers related to glucose metabolism that are altered in AD as opposed to other neurological processes.

Liquid chromatography coupled with tandem mass spectrometry (LC-MS) is becoming a more widely used and efficient method for the unbiased quantification of peptides and proteins in biofluids used in the discovery of novel biomarkers. Previous studies have used MS based techniques to investigate the proteomic landscape of AD in both brain tissue and CSF ^19–24^, highlighting multiple proteomic modules affected in AD, including energy metabolism. With technological advances over the last decade, data-independent acquisition MS (DIA-MS) methods can be employed at a much larger scale than possible in the past, yielding increased depth of proteome coverage with high quantification accuracy ^25–29^. In this study, we performed DIA-MS on CSF collected during a patient’s clinical evaluation in neurology clinic, for suspected AD and a wide variety of other neurological disorders. Age matched cognitively unimpaired control samples were also obtained from clinic attendees. Utilizing a neurology clinic cohort, as opposed to a well-defined high-contrast cohort, more closely aligns with a real-life diagnostic situation, and demonstrates the potential applicability of our findings to a clinical setting.

Here we present two key enzymes involved in glycolysis, Pyruvate Kinase (PKM) and Aldolase A (ALDOA), uncovered by deep proteomic survey of CSF, as potential novel biomarkers for AD. We show that these enzymes are present as full-length proteins in CSF and that levels of tryptic peptides from these enzymes correlate with CSF glucose and lactate levels, but not with a peripheral measure of long-term glucose dysregulation. Further assessment found indications of a similar elevation pattern of other glycolytic markers in AD, such as triosephosphate isomerase (TPI1), alpha and gamma enolase (ENO1; ENO2) in both CSF and publicly available tissue data. The data presented here expand upon previous findings of broad dysregulation of the glucose metabolism machinery in AD and highlight targets for further study.

## Results

### Cohort numbers

**Figure 1:**
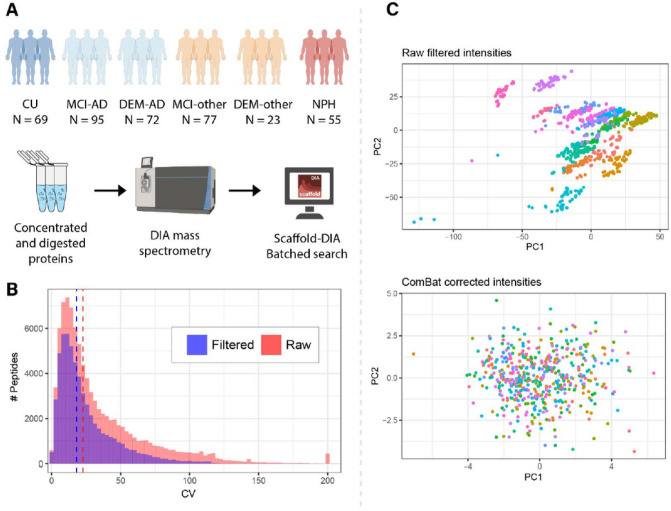
Experimental workflow and MS processing. A) Overview of cohort and sample processing. B) Distribution of duplicate CV before (red) and after (blue) filtering of robustly measured peptides; dotted lines indicate median. C) Principal component analyses before and after ComBat batch correction of the data. Dots represent individual samples and are colored by their corresponding batch.

The cohort analyzed by DIA-MS consisted of 400 different patient samples. The samples in this cohort reflected a diversity of patients from a neurology clinic spanning various non-dementia diagnoses and diagnoses of patients with cognitive impairments of varying degrees of severity. The cohort was selected to reflect a large age range (56–94 years). The samples in the AD groups were defined by a low Ab _42/40_ ratio below 0.0818, to establish AD as the major pathophysiology for dementia, although mixed pathologies could not be ruled out. Nine CU samples were observed to be Ab_42/40_ positive, reflecting “asymptomatic”, “pre-clinical” or “AD resilient” status. These samples were included in the evaluation of the technical variability between injections and batches in MS but excluded from the downstream linear regression (Table 1, Fig. 1A).

### Mass spectrometry search results

Peptide level quantifications from Scaffold-DIA from all batches were combined into one table. To establish a robust dataset, a series of quality control steps were completed. First, a batch-wise pattern of missing values was observed in the combined dataset (**Supplementary Fig. 1**). The dataset was filtered to only keep peptides that were identified and quantified in at least 80% of all samples. Following filtering, the duplicate coefficient of variation (CV) was calculated for pooled samples within each batch. Only the peptides with a mean duplicate CV below 25% were retained, resulting in a final dataset consisting of 2740 unique peptide sequences belonging to 478 unique proteins. By selecting these robustly quantified peptides, the median duplicate CV decreased from 22.8–18.0% (Fig. 1B). Remaining missing values were imputed using the mean intensity value for each peptide. Principal component analyses (PCA) revealed a batch-wise clustering of the samples (Fig. 1C), and the ComBat batch correction algorithm was applied to the dataset ^30^. After batch correction the batch-wise clustering of samples was no longer observed in the PCA (Fig. 1C).

#### Differential abundance analysis.

After establishing a robust dataset of peptide level quantifications in CSF, differential expression of peptides across experimental groups was tested. A linear regression model was fitted to the data and included age and sex as covariates (*peptide intensity* ~ *experimental group + age + sex*). P values were adjusted for multiple-testing using the Benjamini-Hochberg method. In total, there were 2219 significant contrasts between any two groups (adjusted p < 0.05). A high proportion of these contrasts were between NPH and any group. To be more inclusive of potential differences between other non-NPH groups it was decided to include contrasts with a nominal adjusted p-value below 0.1. This yielded 3319 contrasts between any two group, belonging to 1110 unique peptide sequences. The high number of contrasts that were derived from any group compared to NPH reflect a strong differential molecular phenotype of NPH compared to any of the other groups (**Supplementary Fig. 2**). For this reason, to further investigate the molecular differences between the AD and non-AD groups, differential expression in NPH was left out of consideration from subsequent analyses. This resulted in 726 contrasts with a nominal adjusted p-value below 0.1 between any non-NPH group, corresponding to 378 unique peptide sequences.

**Figure 2:**
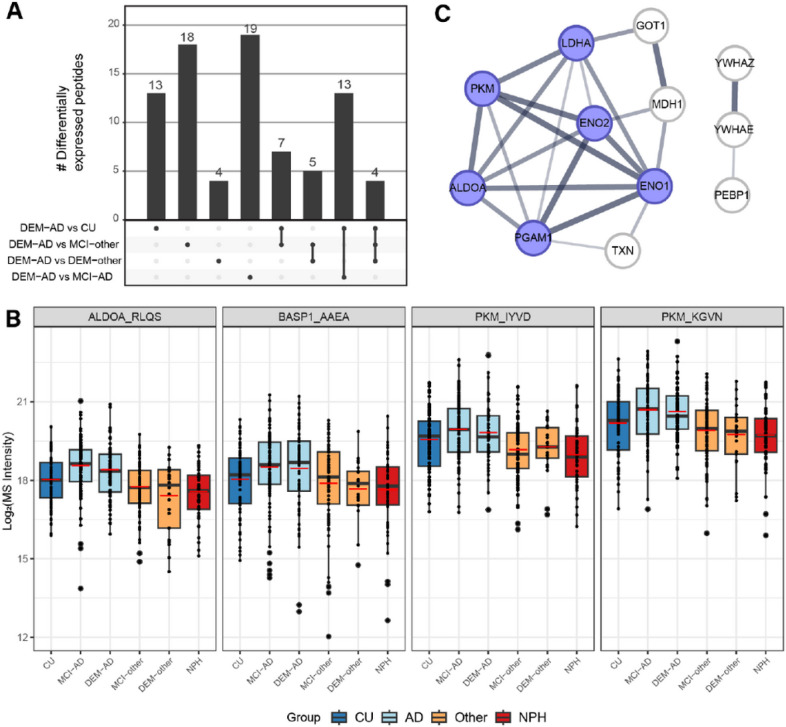
Overview of differentially abundant peptides in CSF in AD. A) Overview of differentially expressed peptides between DEM-AD and any non-AD group. B) MS-intensity levels of 4 peptides differentially expressed in an AD specific pattern (ALDOA: Aldolase-A, BASP1: Brain acid soluble protein 1, PKM: Pyruvate kinase muscle). Red lines indicate mean. C) STRING-DB cluster highlighting the enrichment of proteins involved in energy metabolism in AD CSF. Blue indicates proteins from the GO-term “canonical glycolysis”.

There were 116 contrasts between any diagnostic group compared to DEM-AD that fell below the nominal threshold of adjusted p < 0.1 (Fig. 2A, **Supplementary table 1**). Four peptides were differentially abundant between DEM-AD and all non-AD groups (CU, MCI-other and DEM-other) indicating AD specificity of these peptides (Fig. 2B). These peptides belong to the three proteins Aldolase-A (ALDOA), Pyruvate kinase muscle (PKM) and Brain acid soluble protein-1 (BASP1).

### Protein interaction network

To further explore the proteomic differences, present in CSF in AD, a list of 44 proteins was assembled from the peptides that were found to be differentially abundant between DEM-AD and any other diagnostic group (**Supplementary** table 1). This list was then used to perform a functional network analysis using STRING-DB ^31^. A k-means clustering with three clusters was applied on the network (**Supplementary Fig.3**).

Two of the resulting clusters were functionally annotated to be involved in energy metabolism and neuronal signaling. The energy metabolism cluster included key glycolytic proteins such as ALDOA, PKM, ENO1, ENO2, and phosphoglycerate mutase 1 (PGAM1) (Fig. 2C). It also contained lactate dehydrogenase (LDHA), an enzyme that regulates the conversion of pyruvate into lactate as an alternative endpoint to glycolysis. The neuronal signaling cluster contained multiple well-established synaptic markers such as neuronal pentraxin 1 and 2 (NPTX1; NPTX2) and the neuronal pentraxin receptor (NPTXR) as well as VGF (nonacronymic), which were downregulated in DEM-AD compared to CU and MCI-AD. The third cluster contained apolipoprotein E (APOE), which is genetically associated to AD, and amyloid precursor protein (APP), the parent protein that produces the peptides found in amyloid plaques. APOE was downregulated in DEM-AD compared to CU and MCI-other, where APP was upregulated in DEM-AD compared to MCI-other.

### Protein level validation of mass spectrometry

**Figure 3:**
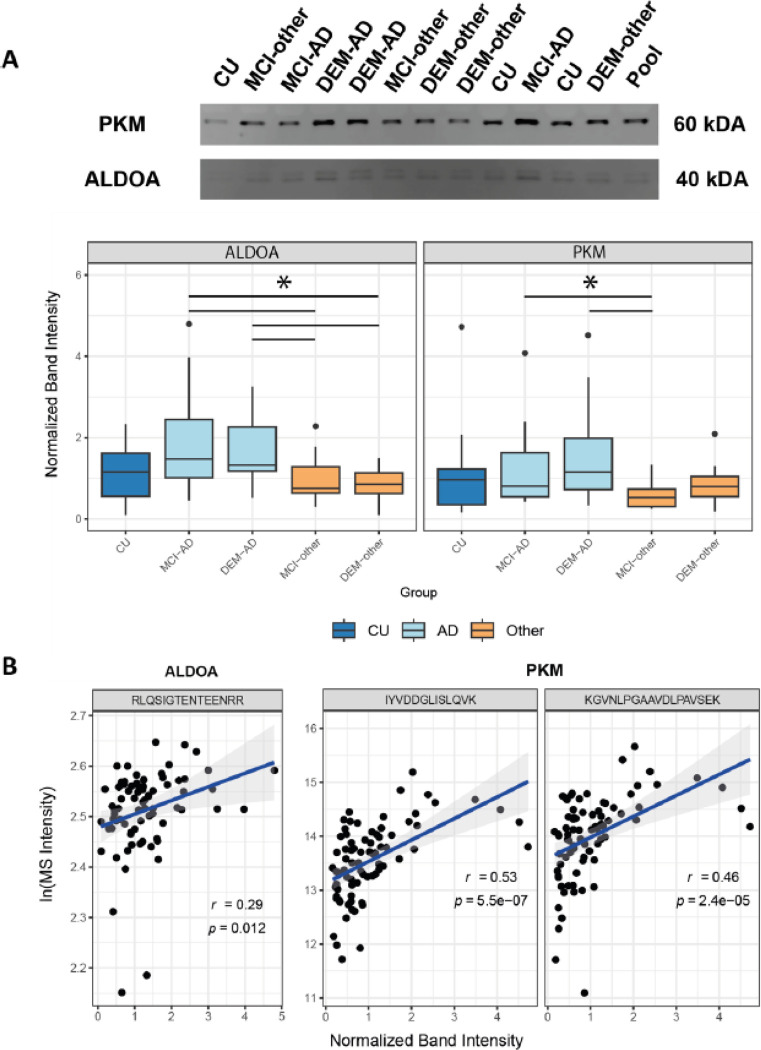
Immunoblotting of ALDOA and PKM indicate the presence of full-length proteins in CSF. A) Normalized band intensities of ALDOA and PKM follow similar intensity pattern across groups as their tryptic peptides (*: p < 0.05). B) MS-intensity of tryptic peptides of both ALDOA and PKM are correlated with the normalized band intensity indicating presence of full-length proteins in CSF.

A balanced subset of the samples that were used for MS was randomly selected to verify the MS peptide level quantifications. The three targets that were specifically elevated in AD compared to other experimental groups, ALDOA, PKM and BASP1, were assessed by Western blot for protein level quantifications. A double banded pattern was detected for ALDOA at the expected 40 kDa size, suggesting the presence of two variants of full-length ALDOA in CSF. For PKM, a single band was detected at the expected 60 kDa size indicating presence of full-length protein in CSF (Fig. 3A). For BASP1 multiple bands were detected both at the expected size and other sizes, indicating the likely presence of multiple post translational processing products.

Relative band intensity compared to a pooled sample was measured across diagnoses for all three targets (Fig. 3A; **Supplementary Fig. 4A**). For both ALDOA and PKM, the normalized band intensities followed a similar abundance pattern to their tryptic peptides in MS and non-parametric Kruskall-Wallis tests indicated significant differences between groups. For ALDOA, post-hoc Dunn-test showed that both MCI-AD and DEM-AD band intensities were found to be significantly increased compared to both MCI-other and DEM-other (p < 0.05). PKM normalized band intensities were significantly elevated in both MCI-AD and DEM-AD compared to MCI-other (p < 0.05). BASP1 normalized intensities of bands at the expected kDa range did not follow the peptide abundance pattern observed from MS data (**Supplementary Fig. 4B**).

To further establish that the measured MS-intensities for these markers reflect the levels of full-length proteins in CSF, correlations between normalized band intensity and MS-intensity for the individual tryptic peptides belonging to ALDOA and PKM was determined (Fig. 3B). For both tryptic peptides of PKM a significant positive correlation was observed (r = 0.53, p = 5.5×10^−7^; r = 0.46, p = 2.4×10^−5^). For the tryptic peptide of ALDOA a significant moderate positive correlation was observed (r = 0.29, p = 0.012).

### Changes in metabolic markers in CSF and periphery

**Figure 4:**
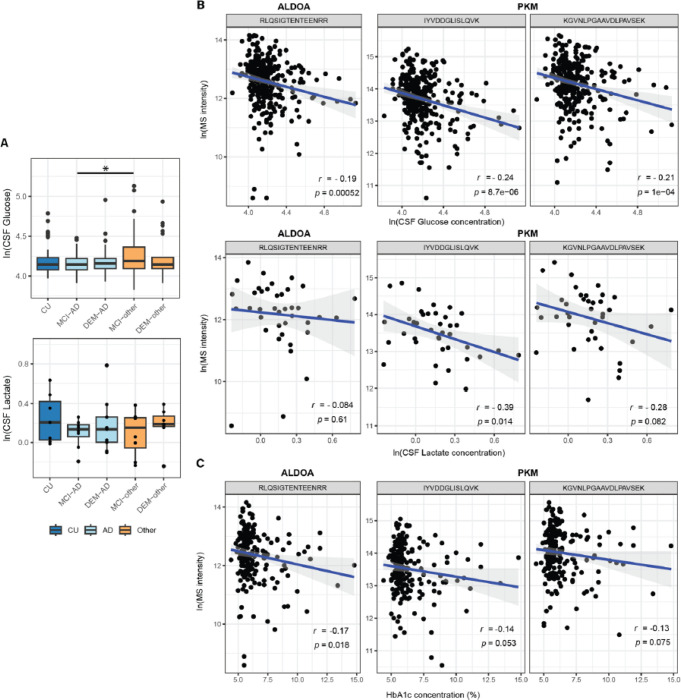
Measures of metabolites in CSF. A) Levels of glucose and lactate in CSF across groups. (*: p < 0.05) B) MS intensities for ALDOA and PKM peptides correlate negatively with the CSF glucose and lactate levels. C) Peripheral glucose metabolism marker HbA1c did not show a strong correlation with MS intensities for ALDOA and PKM peptides.

Following the potential indication of altered glucose metabolism in the CSF of AD patients, the levels of selected metabolites in the same CSF samples were investigated. In a group level comparison, CSF glucose levels were only elevated in MCI-other compared to MCI-AD (p < 0.05), but not in any other contrast. No significant changes in lactate levels were observed between groups (Fig. 4A). Correlation between MS-intensity for the AD-specific tryptic peptides of ALDOA and PKM with levels of glucose and lactate was investigated (Fig. 4B). CSF glucose levels were found to be significantly negatively correlated with the MS-intensity of the tryptic peptides of both ALDOA (r = −0.19, p = 5.2×10^−4^) and PKM (r = −0.24, p = 8.7×10^−6^; r = −0.21, p = 1.0×10^−4^). One tryptic peptide of PKM (*IYVDDGLISLQVK*) correlated significantly with the CSF lactate levels (r = −0.39, p = 0.014). Additionally, the relationship of these tryptic peptides with Hemoglobin A1C (HbA1c), a measure of long-term peripheral glucose dysregulation, was investigated (Fig. 4C). ALDOA (*RLQSIGTENTEENRR*) MS intensity showed a modest but significant negative correlation (r = −0.17, p = 0.018) with HbA1C.

### Levels of glycolysis markers in CSF and tissue

**Figure 5:**
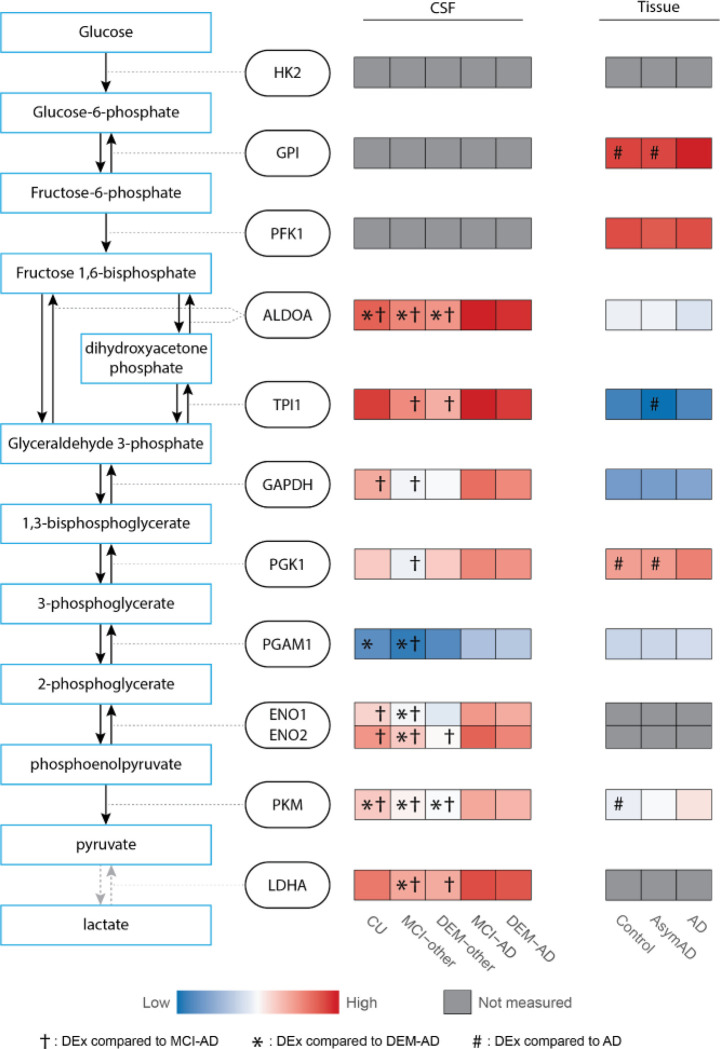
Levels of glycolytic enzymes are generally elevated in AD in CSF and brain tissue. Colors represent scaled peptide or protein abundance across all proteins for each individual dataset. †: DEx compared to MCI-AD, *: DEx compared to DEM-AD, #: DEx compared to AD in tissue.

With the AD specific elevation of the two key glycolytic enzymes, ALDOA and PKM, we investigated changes in the levels of other glycolytic enzymes. The first three enzymes in glycolysis, hexokinase (HK2), glucose-6-phosphate isomerase (GPI) and phospho-fructokinase (PFK1), were not robustly measured in CSF in this analysis. The other enzymes, TPI, glyceraldehyde-3-phosphate dehydrogenase (GAPDH), phosphoglycerate kinase (PGK1), PGAM1, ENO1, and ENO2, were all decreased in one or more non-AD group compared to either MCI-AD or DEM-AD (adjusted p-value < 0.1; Fig. 5). LDHA was also found to be upregulated in AD compared to the non-AD dementia groups.

To gain insight into the possible source of the changes of glycolytic enzymes in CSF, publicly available protein abundance data from brain tissue was investigated ^19^. Here, AD brain tissue from the dorsolateral prefrontal cortex was compared to tissue from healthy controls and subjects without dementia but with postmortem AD pathology (AsymAD). In Fig. 5 we plot the mean scaled abundances for each group using the author’s significance values generated from post hoc Tukey tests (Fig. 5). The enzymes, GPI, PGK1 and PKM were elevated in AD compared to controls (p < 0.05). GPI, PGK1 and TPI1 were elevated in AD compared to AsymAD (p < 0.05), suggesting their elevation is related to cognitive dysfunction as opposed to amyloid pathology. The enzymes HK2, ENO1 and ENO2, and LDHA were not robustly measured in the brain tissue, as defined by being quantified in less than 50% of all samples. Although the changes of specific glycolytic enzymes do not necessarily correspond between tissue and CSF, a general elevation of multiple glycolytic enzymes in both matrices indicate general disturbance of glycolysis in the AD brain.

## Discussion

Heterogeneity amongst AD patients necessitates the stratification of patients to specific molecular targets for effective therapeutic intervention. The complex and heterogenous nature of AD and its intersecting pathophysiologies has made it difficult to specify these targets. Here, we present a deep CSF proteomic survey of a large clinically diverse patient cohort. Differential expression indicated a strong enrichment of glucose metabolism markers in AD compared to non-AD groups. Specifically, four peptides were found to be elevated following an AD-specific expression pattern belonging to two glycolytic enzymes ALDOA and PKM, and a neuronal membrane protein BASP1. Through immunoblotting, both ALDOA and PKM, but not BASP1 were verified to be present as full-length proteins in CSF. Levels of tryptic peptides derived from these glycolytic enzymes correlated with glucose and lactate levels in the corresponding CSF sample. Finally, we showed that other glycolytic enzymes follow a similar AD-specific elevation pattern in CSF and brain tissue homogenate, although there are sample type specific effects.

Altered glucose metabolism in the brain has long been observed as a key step in the development of AD. Brain hypometabolism as measured by FDG-PET is a central biomarker of disease, however the exact neurobiological source of these changes remains unclear ^32–34^. Previous studies demonstrated changes in proteins involved in energy metabolism in CSF from patients with AD; specifically, both PKM and ALDOA were upregulated in AD diagnoses (Higginbotham et al., 2020; Johnson et al., 2020). Our findings validate these reports and indicate, through immunoblotting, that both ALDOA and PKM are present as full-length functional proteins in CSF. Presence of full length BASP1 in CSF could not be confirmed through immunoblotting. It is a 23 kDa protein with an electrophoresis mobility of 58 kDa ^35,36^. The quantification of BASP1 through immunoblot is made more challenging by the presence of multiple bands, probably deriving from non-tryptic peptide products, that occur at different predicted kDa heights depending on SDS percentage used. The potential functionality of these non-tryptic peptide products should be studied further.

ALDOA has previously been associated with AD and other neurological disorders ^37,38^, including identification as a possible autoantigen in AD ^38^. While overlapping pathology between AD and other groups suggested ALDOA was a non-specific biomarker for AD and thus a poor candidate for immunotherapy ^37,39^, the utility of ALDOA as a biomarker for stratification within defined AD patients has not been fully explored.

PKM is a critical rate-limiting enzyme in glycolysis that regulates the generation of pyruvate, and as such it has been widely studied in cancer cells, a cell type with high metabolic activity ^40^. Aerobic glycolysis occurs when pyruvate is processed through the tricarboxylic cycle, resulting in a high energy yield. By contrast, tumor cells anaerobically convert pyruvate into lactate via LDHA in a phenomenon termed the Warburg effect ^40^. The changes in neuroenergetics occurring in AD have previously been described as an inverse Warburg effect ^41,42^. In an AD patient-derived induced neuronal model, it was shown that one specific PKM isoform, PKM2, regulates an anaerobic glycolytic shift, similar to the Warburg effect, whereas PKM1 regulates aerobic glycolysis ^43^. A PKM2-specific targeting anti-cancer drug was shown to ameliorate this effect. PKM2, has also been directly linked to Ab plaque promotion by positively regulating gamma-secretase in the cytosol in hypoxic conditions ^44^. The peptides identified by DIA in this study could not distinguish between the PKM1 and PKM2 isoforms, and western blotting with a PKM2 specific antibody in CSF samples showed no quantifiable signal (data not shown). However, the results presented here potentially indicate that the changes in PKM levels we observe in patient-derived CSF may be related to the changes described by Traxler *et al.*, (2022) who show widespread increases in most glycolytic enzymes in response to elevation of metabolically inactive PKM2. The results presented here indicate specific elevation of ALDOA and PKM proteins in AD CSF related to changes in glycolysis, indicating broad dysregulation of glucose metabolism in the AD brain and highlighting both as a potential therapeutic target protein.

### Limitations

Despite the technological advances in DIA-MS that allow for experiments on large numbers of samples, the downstream analysis of such experiments is still a subject of ongoing development. Analyses on a large-scale cohort using Scaffold-DIA, as presented here, required both a batch-wise division in MS scanning and subsequent downstream analyses due to computational limitations of analysis software. Despite the deep scanning power of DIA-MS, normally leading to very low numbers of missing values in its peptide identification, the batch-wise approach applied here lead to a considerable number of missing values. With advances in the computational tools required for MS identifications, a more comprehensive peptide identification search of all batches together is expected to lead to less missingness. Additionally, advances in batch-correction methods specifically developed for MS data is expected to improve the analyses of such large-scale datasets ^45,46^. To overcome these issues in this study, very robust cutoffs were applied when curating the dataset, resulting in high confidence on the identification and quantitation of the peptides presented here. However, this could implicate that some informative peptides were excluded from our analyses. Furthermore, this analysis only included peptides originating from tryptic digests. Peptides derived from non-tryptic cleavages have been shown to be present and active in the brain and CSF ^47^. Expanding the peptide identification search to include peptides from non-tryptic cleavages in CSF would create a more representative picture of the molecular landscape but would significantly increase the computing capacity needed for such analyses ^48,49^. Although the large sample in this study yields a better understanding of a real-world clinically diverse cohort, it is limited by the absence of longitudinal cognitive data and samples. Measurements of better biomarkers over time are imperative to better describe disease progression from early stages to further developed disease ^50^.

In conclusion, this study applied DIA-MS on CSF from a large-scale clinically complex cohort, confirming previous findings of dysregulated glucose metabolism in AD. We find the AD-specific upregulation of two glycolytic enzymes, ALDOA and PKM, and highlight these enzymes as putative biomarkers for impaired brain metabolism in the AD brain.

## Methods

### Study cohort

CSF samples were obtained according to standardized collection and processing protocols through the Mass General Institute for Neurodegenerative Disease biorepository, following written informed consent for research biobanking (IRB: 2015P000221). This repository consists of CSF samples from diagnostic lumbar punctures at the Department of Neurology at Massachusetts General Hospital. CSF levels of Aβ_1_ − _40_, Aβ1 − 42, pTau (181) and total tau (tTau) were measured by commercially available ELISA assays (Euroimmun, Lubeck, Germany). Clinical diagnoses were established through clinical review by an experienced neurologist (SEA) and AD status corroborated with CSF AD biomarkers showing low Aβ42/40 ratio using lab derived cut-points. Samples were subdivided into six groups based on clinical diagnosis: cognitively unimpaired (CU; n = 69), mild-cognitively impaired AD (MCI-AD; n = 95), mild-cognitively impaired from other causes (MCI-other; n = 77), dementia AD (DEM-AD; n = 72), dementia from other causes (DEM-other; n = 23) and Normal Pressure Hydrocephalus, based on positive “tap-test” gait outcome after large volume lumbar puncture (NPH; n = 55) (Table 1). Cognitively unimpaired individuals with CSF A-beta positivity (n = 9) were excluded from the study.

### CSF processing

Samples were processed for LC-MS/MS by investigators blinded to clinical status. Aliquots were frozen and stored at −80°C in low-binding polypropylene tubes and thawed on ice for use. 200 μL samples, when possible, were dried in a SpeedVac, and resolubilized in 100 μL 8 M urea/0.4 M ammonium bicarbonate, or half the original volume for low volume aliquots. Protein concentration was measured through Pierce BCA assay (Thermo Fisher Scientific, Waltham MA, USA) and samples were adjusted to 7.5 μg protein in 50 μL. Samples were reduced with a 1:10 dilution of 45 mM DTT and incubated at 37°C for 30 min. Subsequently, samples were alkylated with a 1:10 dilution of 100 mM Iodoacetamide at room temperature in the dark for 30 min Urea concentration was then lowered to 2 M using water. Proteins were digested overnight at 37°C with 1:20 LysC:sample protein followed by an additional 1:20 trypsin digestion for 8 hours at 37°C. Samples were then acidified with 20% TFA and desalted using Nest Group C18 macrospin columns (HMMS18V) following the manufacturer’s instructions, and the eluent was dried for mass spectrometry.

### Data-Independent Acquisition

DIA LC–MS was performed using a nanoACQUITY UPLC system (Waters Corporation, Milford, MA, USA) connected to a Thermo Orbitrap Fusion mass spectrometer (ThermoFisher Scientific, San Jose, CA, USA). Samples were resuspended in dH_2_O with 2% acetonitrile and 0.2% TFA and injected in batches of 24 at a time, with each sample injected in duplicate and a pooled control sample at the start and end of each batch. After injection, the samples were loaded into a trapping column (Waters ACQUITY UPLC M-Class Symmetry^®^ C18 trap column, 5 micro, 180 μm × 20 mm) at a flow rate of 5 μL/min and separated with an analytical column (Waters ACQUITY UPLC M-Class Peptide BEH C18 column, 1.7 micro, 75 μm × 250 mm). The compositions of mobile phases A and B were 0.1% formic acid in water and 0.1% formic acid in acetonitrile, respectively. The peptides were separated and eluted with a 120-min gradient extending from 6–35% mobile phase B in 85 min and then to 85% mobile phase B in additional 5 min at a flow rate of 300 nL/min and a column temperature of 37°C. Column regeneration and up to three blank injections were carried out in between all sample injections. The data were acquired with the mass spectrometer operating in a Data-Independent Acquisition mode with an isolation window width of 25 m/z. The full scan was performed in the range of 400–1,000 m/z with “Use Quadrupole Isolation” enabled at an Orbitrap resolution of 120,000 at 200 m/z and automatic gain control target value of 4 × 10^5^. Fragment ions from each MS^2^ non-overlapping isolation window were generated in the C-trap with higher-energy collision dissociation at a collision energy of 28% and detected in the Orbitrap at a resolution of 60,000.

Using Scaffold DIA software v. 2.2.0 (Proteome Software, Portland, OR, USA), DIA spectra were searched, per batch, against a *Homo Sapiens* CSF proteome spectral library generated from a collection of DDA LC MS/MS spectra acquired from an offline UPLC fractionated pooled CSF sample on the same Thermo Orbitrap Fusion mass spectrometer. Within Scaffold DIA, raw DIA data files were first converted to the mzML format using ProteoWizard v. 3.0.11748. The samples were then aligned by retention time and individually searched against the spectral library with a peptide mass tolerance of 10 ppm and a fragment mass tolerance of 10 ppm. The data acquisition type was set to “Non-Overlapping DIA”, and the maximum missed cleavages was set to 2. Fixed and dynamic modifications included carbamidomethylation of cysteine (+ 57.02) and oxidation of methionine (+ 15.99), respectively. Peptides were initially filtered by Percolator v. 3.01 at a threshold FDR of 0.01. Resulting peptides with charge states between 2 and 4 with peptide lengths of 6–30 amino acids residues were considered for quantitation. Peptide quantification was performed by EncyclopeDIA v. 0.9.6 and five of the highest quality retention time aligned fragment ions were selected for quantitation. The peptide level quantifications from the batched Scaffold-DIA searches were analyzed in R.

### Immunoblot confirmation

A random balanced subset of samples from the mass spectrometry cohort was selected as a validation cohort. Samples were spun down at 3,000× g for 10 min and the resulting supernatant was used for immunoblotting. Total sample protein concentration was determined using the Pierce BCA assay and 20 μg of protein per sample was loaded on the gel. A pooled sample was generated from the samples and loaded on each gel for between-blot normalization. Novex Wedgewell 4–20% polyacrylamide gels (Invitrogen, Waltham MA, USA) were used, and samples were run at 55 mA per gel. Subsequently, samples were transferred to nitrocellulose membrane at 300 mA for 90 min (Bio-Rad laboratories, Hercules CA, USA). After transfer, membranes were blocked for 1 hour using LI-COR PBS intercept blocking buffer. Primary antibody dilutions were prepared using the LI-COR Intercept T20 PBS antibody diluent. Antibodies for ALDOA (D73H4, Cell Signaling Technology) was used at a concentration of 1:500, PKM (C103A3, Cell Signaling Technology) at 1:1000 and BASP1 (5H3L1, ThermoFisher) at 1:500. Membranes were incubated overnight in primary antibody at 4°C. Membranes were then washed 4x in PBS-T and incubated for 1 hour in LI-COR goat anti-rabbit secondary antibody diluted 1:1000 in LI-COR Intercept T20 PBS antibody diluent. Blots were imaged with a LI-COR CLx imager and band intensities compared to background were measured using the Image Studio Light and ImageJ.

### CSF glucose and lactate measurement

CSF glucose levels were assessed in the Pathology Core at Massachusetts General Hospital through clinical standard enzymatic hexokinase assay at the same time as the CSF collection. HbA1c measurements were obtained through electronic health records from matching patients where available. For patients with multiple HbA1c measurements, the highest value was used for analysis. Lactate concentration was measured on a randomly selected balanced subset of matched samples through enzymatic assay (ab65331, Abcam), according to the manufacturer’s protocol. Samples were diluted 12x to fall within the detectable range. Total glucose and lactate levels between diagnostic groups was determined and concentrations were correlated to the MS intensity of the individual tryptic peptides of ALDOA and PKM.

### Statistical analysis

All data analysis was performed in R-studio under R version 4.2.2. Raw MS intensities were imported from Scaffold-DIA and processed as described in results. Pattern of missingness was determined by counting the number of samples per peptide where that peptide was not quantified. Batch correction was performed using the *ComBat()* function from the sva package (version 3.46.0). Linear modeling of the MS data was performed using the *lm()* function and the results were tidied up using the *tidy()* function from the broom package (version 1.0.4). Correlations were calculated with Pearson correlation methods using the *cor.test()* function. Between group differences for immunoblotting and metabolite levels were calculated using non-parametric Kruskall-Wallis test using *Kruskal.test()* and post-hoc analyzed with *dunnTest()* from the FSA package (version 0.9.4)

## Data Availability

Normalized MS intensity values of all peptides of glycolytic proteins can be found in supplementary table 2. Other underlying data will be made available upon request to the corresponding author.

## Abbreviations

Ab: Amyloid-beta
ALDOA: Aldolase
AD: Alzheimer’s disease
APOE: Apolipoprotein E
APP: Amyloid precursor protein
AsymAD: Subject without dementia but with postmortem AD pathology
BASP1: Brain acid soluble protein-1
CSF: cerebrospinal fluid
CV: Coefficient of variation
CU: Cognitively unimpaired
DEM-AD: Dementia AD
DEM-other: Dementia from other causes
DIA-MS: Data-independent acquisition MS
ENO1; ENO2: Alpha and gamma enolase
FDG-PET: Fluorodeoxyglucose positron emission tomography
GAPDH: Glyceraldehyde-3-phosphate dehydrogenase
GPI: Glucose-6-phosphate isomerase
HbA1c: Hemoglobin A1C
HK2: Hexokinase
LC-MS: Liquid chromatography - tandem mass spectrometry
LDHA: Lactate dehydrogenase
MCI-AD: Mild-cognitively impaired AD
MCI-other: Mild-cognitively impaired from other causes
NPH: Normal Pressure Hydrocephalus
NPTX1; NPTX2: Neuronal pentraxin 1 and 2
NPTXR: Neuronal pentraxin receptor
PCA: Principal component analyses
PFK1: Phospho-fructokinase
PGAM1: Phosphoglycerate mutase 1
PGK1: Phosphoglycerate kinase
PHFtau: Paired helical filaments of hyperphosphorylated tau
PKM: Pyruvate kinase
TPI1: Triosephosphate isomerase
